# Adrenalectomy Improves Muscle Strength in Patients with Mild Autonomous Cortisol Secretion: A Comparative Study

**DOI:** 10.3390/medicina62030553

**Published:** 2026-03-16

**Authors:** İsmail Engin, Mazhar Müslüm Tuna

**Affiliations:** Department of Endocrinology and Metabolism, Health Sciences University Ümraniye Training and Research Hospital, Istanbul 34760, Turkey

**Keywords:** mild autonomous cortisol secretion, MACS, adrenal incidentaloma, adrenalectomy, muscle strength, hand grip strength, sarcopenia

## Abstract

*Background/Objectives*: Mild autonomous cortisol secretion (MACS) is increasingly recognized in patients with adrenal incidentalomas. While associated with cardiometabolic risk, management strategies remain controversial, particularly regarding functional outcomes. This retrospective comparative cohort study evaluated the impact of adrenalectomy versus conservative management on muscle strength in MACS patients. *Materials and Methods*: Forty patients with MACS (1-mg dexamethasone suppression test > 1.8 µg/dL) were enrolled: 15 underwent adrenalectomy and 25 received conservative management. Hand grip strength was measured using calibrated dynamometry, and gait speed was assessed with the 1-m walk test at baseline and 6 months. *Results*: Baseline characteristics are summarized descriptively for the surgical and conservative cohorts. At 6 months, the surgery group showed significant improvements in right hand grip strength (+1.19 ± 0.64 kg, *p* < 0.001) and left hand grip strength (+1.15 ± 0.49 kg, *p* < 0.001), representing approximately 5% improvement. In contrast, the conservative group exhibited significant decreases in strength over the same period (right: −0.40 ± 0.25 kg; left: −0.28 ± 0.28 kg, both *p* < 0.001). The post-surgical 1-mg dexamethasone suppression test decreased from 4.05 ± 1.44 to 1.01 ± 0.34 µg/dL (*p* < 0.001). *Conclusions*: Adrenalectomy results in significant improvement in objective muscle strength in MACS patients, with improvement observed in parallel to biochemical resolution of cortisol excess. In contrast, conservative management was associated with progressive decline in grip strength over 6 months. Hand grip dynamometry provides valuable functional outcome data that may guide surgical decision-making in MACS management.

## 1. Introduction

Mild autonomous cortisol secretion (MACS), formerly known as subclinical Cushing’s syndrome, is increasingly recognized in patients evaluated for adrenal incidentalomas, with a prevalence ranging from 5% to 30% depending on diagnostic criteria [1,2]. While MACS is characterized by cortisol excess without the overt clinical features of Cushing’s syndrome, accumulating evidence demonstrates its association with increased cardiometabolic morbidity, including hypertension, diabetes, dyslipidemia, and reduced bone mineral density [3,4].

Despite these associations, the optimal management strategy for MACS remains controversial. The 2016 European Society of Endocrinology guidelines and the 2021 AACE/AAE position statement acknowledge the heterogeneity of MACS presentation and recommend individualized treatment decisions [1,5]. While adrenalectomy has been shown to improve some metabolic outcomes and bone density, data on functional outcomes particularly muscle strength and physical performance remain limited.

Our cohort met current ESE 2023 diagnostic criteria for MACS, with appropriately suppressed ACTH levels (mean < 10 pg/mL in both groups) and DHEA-S levels documented as part of comprehensive hormonal characterization. The baseline hormonal profile including both dexamethasone suppression test (DST) results and ACTH suppression confirmed the MACS diagnosis and excluded overt Cushing’s syndrome. While no marked differences were identified in DHEA-S suppression between surgical and conservative groups (*p* = 0.596), the documentation of these parameters aligns with ESE 2023 emphasis on comprehensive hormonal characterization beyond DST alone. Lower DHEA-S levels are known to accompany MACS and may be independently associated with reduced muscle function in older adults, providing biological plausibility for investigating muscle outcomes in this population.

Glucocorticoid excess is well-known to cause skeletal muscle atrophy through multiple mechanisms, including increased protein catabolism, decreased protein synthesis, and mitochondrial dysfunction [6,7]. Although patients with overt Cushing’s syndrome frequently present with proximal muscle weakness, the extent and clinical significance of myopathy in MACS have remained poorly characterized in the literature. Muscle weakness is often reported as a complaint by patients with MACS, yet objective assessments of muscle strength using standardized tools such as hand grip dynamometry are rarely documented in prospective clinical studies.

Hand grip strength measurement is a simple, reliable, and validated office-based assessment that correlates with overall muscle strength, functional capacity, and health outcomes [8,9]. Similarly, gait speed represents an established functional measure that predicts morbidity and mortality in older adults [10]. Whether surgical management of MACS can improve these objective functional parameters remains an important clinical question, particularly when weighing the benefits and risks of adrenalectomy in individual patients.

Therefore, we conducted this retrospective comparative cohort study to compare the effects of adrenalectomy versus conservative management on objective muscle strength (hand grip strength) and functional performance (gait speed) in patients with MACS. We hypothesized that surgical correction of cortisol excess would result in measurable improvements in muscle strength, with these improvements parallel to biochemical resolution of the hypercortisolemic state.

## 2. Materials and Methods

### 2.1. Study Design and Participants

This study was designed as a retrospective comparative cohort study based on the analysis of clinical records. The current analysis was conducted retrospectively using data obtained from January 2023 to September 2024. The study included patients for whom hand dynamometry and gait speed data were available as part of their routine outpatient and inpatient clinical evaluations.

Patients aged 18–75 years with adrenal incidentalomas (>1 cm) and biochemical evidence of MACS were eligible for enrollment. MACS is characterized by serum cortisol levels exceeding 1.8 µg/dL following a 1-mg overnight dexamethasone suppression test, in the absence of pronounced Cushingoid characteristics [1,5]. Exclusion criteria included: (1) overt Cushing’s syndrome with typical clinical features; (2) other functioning adrenal tumors (pheochromocytoma, aldosteronoma); (3) malignant adrenal lesions; (4) severe musculoskeletal or neurological disorders affecting grip strength; (5) recent fracture or surgery of upper extremities; and (6) inability to complete follow-up assessments.

Assignment to surgical versus conservative management was based on clinical indication and shared decision-making between patient and physician. Surgical intervention was generally recommended for patients with: (1) larger tumor size (>4 cm); (2) imaging characteristics suspicious for malignancy; (3) progressive metabolic complications; (4) worsening hypertension or diabetes control; or (5) young age with long life expectancy. Patients who opted for conservative management were observed without changes to their antihypertensive or antidiabetic medications during the study period, as all patients had stable, optimized medical regimens for at least 3 months prior to baseline assessment.

### 2.2. Surgical Procedure and Perioperative Management

All adrenalectomies were performed via laparoscopic transperitoneal approach by experienced endocrine surgeons. Patients received standard preoperative assessment and routine postoperative care. No intraoperative glucocorticoid supplementation was required in any operated patient.

Postoperative glucocorticoid replacement: Based on postoperative morning serum cortisol assessment, five patients required glucocorticoid replacement. Hydrocortisone was administered at a physiologic replacement dose of 12 mg/m^2^/day (body surface area-adjusted). Replacement was continued for an average of 30 days and then tapered gradually until discontinuation.

### 2.3. Muscle Strength and Functional Assessment

Patients were seated with shoulders adducted, elbow flexed at 90°, and forearm in neutral position as per standardized protocol [11]. The measurement procedure included: (1) one familiarization trial to ensure proper technique, (2) three consecutive measurements for each hand (right, then left) with 30-s rest intervals, and (3) the mean of the three trials was recorded. Dominant hand (right vs. left) and handedness (right-handed vs. left-handed) were documented for all participants to contextualize grip strength differences. Walking speed (m/s) was calculated as 1.0/time (s) for the 1-m walk test.

Gait speed was assessed using the 1-m walk test, a validated measure of functional mobility [10]. Patients were asked to walk at their usual pace over a marked 1-m distance, and time was recorded to the nearest 0.01 s. The test was performed twice, and the mean was calculated.

### 2.4. Biochemical Assessment

MACS was defined as a suppression test result > 1.8 µg/dL of 1 mg overnight dexamethasone in the absence of significant clinical signs of Cushing’s syndrome, according to the 2023 European Society of Endocrinology guidelines. Patients with suppressed or low-normal ACTH levels (<10 pg/mL) were evaluated according to ESE 2023 criteria. DHEA-S levels and their suppression relative to age- and sex-matched norms were documented as part of comprehensive hormonal characterization. It was confirmed that patients were not using medications known to affect dexamethasone metabolism (e.g., phenytoin, rifampin, mitotan), and potential confounding factors were considered at the time of diagnosis.

Baseline hormonal evaluation included morning (08:00) serum cortisol, ACTH, DHEA-S, and 1-mg overnight dexamethasone suppression test. At 6-month follow-up, the surgery group underwent repeat 1-mg dexamethasone suppression testing to confirm biochemical resolution. All biochemical analyses were performed at the hospital’s central laboratory using standardized assays.

### 2.5. Statistical Analysis

For within-group changes over time (baseline to 6-month), paired *t*-tests were used to determine whether each group exhibited statistically significant changes in grip strength and 1-m walk test performance. Pre-specified direct between-group hypothesis testing was not performed because the cohorts were imbalanced in size and demonstrated heterogeneous baseline distributions, which was judged to limit interpretability and reduce the likelihood of detecting meaningful differences with simple unadjusted intergroup comparisons in this retrospective setting. Accordingly, analyses focused on within-group trajectories over time.

Continuous variables are presented as mean ± standard deviation for normally distributed data or median (interquartile range) for non-normally distributed data. Categorical variables are presented as frequencies and percentages.

A two-sided *p*-value < 0.05 was considered statistically significant for within-group analyses. All analyses were performed using SPSS version 25.0 (IBM Corp., Armonk, NY, USA).

To address the between-group comparison—the primary research question of this study—three complementary analyses were additionally performed: independent samples *t*-test comparing change scores (post−pre) between groups, with normality confirmed by Shapiro-Wilk test; analysis of covariance (ANCOVA) with post-treatment value as the dependent variable and baseline value and age as covariates; and Cohen’s d effect size estimation. Mann-Whitney U test was computed as non-parametric corroboration.

## 3. Results

### 3.1. Baseline Characteristics

A total of 40 patients with MACS were enrolled: 15 in the surgical group and 25 in the conservative management group. Baseline characteristics are summarized in Table 1.

Prevalence of comorbidities was high in both groups, with no significant differences in diabetes (40% vs. 40%, *p* = 1.000), hypertension (60% vs. 64%, *p* = 0.805), cardiovascular disease (53% vs. 36%, *p* = 0.281), or hyperlipidemia (33% vs. 36%, *p* = 0.867). Osteoporosis was numerically more common in the surgery group but did not reach statistical significance (33% vs. 20%, *p* = 0.350).

Baseline hormonal parameters are presented descriptively in Table 1. Morning cortisol, ACTH, DHEA-S, and post-1-mg dexamethasone suppression test values are reported for both cohorts to characterize MACS status at study entry.

Baseline muscle strength and gait test measurements are presented descriptively in Table 1 for both cohorts.

### 3.2. Changes in Muscle Strength and Gait Speed

**Figure 1:**
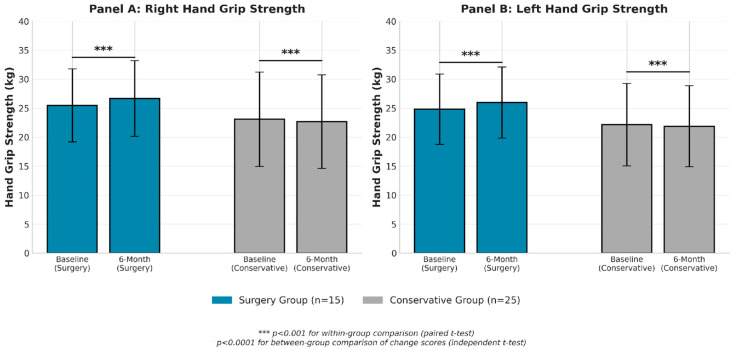
Changes in Hand Grip Strength from Baseline to 6 Months. Bar graphs showing mean (±SD) right hand grip strength (Panel **A**) and left hand grip strength (Panel **B**) at baseline and 6 months for surgical and conservative management groups. *** *p* < 0.001 for within-group comparison (paired *t*-test).

At 6-month follow-up, the surgical group demonstrated significant improvements in bilateral hand grip strength (Table 2, Figure 1). Right hand grip strength improved from 25.49 ± 6.31 kg at baseline to 26.68 ± 6.53 kg at 6 months, indicating a mean enhancement of 1.19 ± 0.64 kg (4.7% increase, *p* < 0.001). Left hand grip strength improved from 24.83 ± 6.07 kg to 25.98 ± 6.13 kg, reflecting a mean enhancement of 1.15 ± 0.49 kg (4.6% increase, *p* < 0.001).

In contrast, the conservative management group experienced small but statistically significant decreases in grip strength. Right hand grip strength decreased by 0.40 ± 0.25 kg (from 23.09 ± 8.14 to 22.69 ± 8.07 kg, *p* < 0.001), and left hand grip strength decreased by 0.28 ± 0.28 kg (from 22.16 ± 7.10 to 21.88 ± 6.99 kg, *p* < 0.001).

Baseline comparison between groups revealed that the surgical and conservative cohorts differed significantly in grip strength and 1-m walk speed at baseline (Table 3), underscoring the need for covariate-adjusted analyses.

Within-group analysis using paired *t*-tests showed that grip strength improved significantly in the surgical group (*p* = 0.002) and declined significantly in the conservative group (*p* < 0.001), whereas 1-m walk time did not change significantly in the surgical cohort (*p* = 0.820) (Table 4).

Between-group comparison of change scores confirmed that the surgical cohort demonstrated a significantly greater improvement in grip strength (Δ = +0.719 kg) compared with the conservative cohort (Δ = −0.382 kg; t = 4.919, *p* < 0.001, Cohen’s d = 1.56, large effect), while between-group differences in 1-m walk did not reach statistical significance (*p* = 0.063) (Table 5).

ANCOVA adjusted for baseline value and age confirmed the between-group differences: adrenalectomy was independently associated with significantly higher post-treatment grip strength and lower (faster) post-treatment walk time compared with conservative management (Table 6).

### 3.3. Biochemical Outcomes

All patients in the surgical group achieved biochemical resolution of cortisol excess. The post-1-mg dexamethasone suppression test decreased from 4.05 ± 1.44 µg/dL at baseline to 1.01 ± 0.34 µg/dL at 6 months (*p* < 0.001), thereby confirming the successful treatment of MACS. This biochemical improvement paralleled the observed gains in muscle strength.

## 4. Discussion

This retrospective comparative cohort study demonstrates that adrenalectomy is associated with significant and measurable improvements in objective muscle strength in patients with MACS. At 6 months post-surgery, patients showed approximately 4.7% improvement in bilateral hand grip strength, parallel to biochemical resolution of cortisol excess. In contrast, conservatively managed patients experienced significant decreases in muscle strength over the same period. These findings suggest that hand grip dynamometry, a simple and inexpensive office-based assessment tool, may provide useful functional outcome data to support clinical decision-making in MACS patients, particularly those reporting muscle weakness.

The pathophysiology of glucocorticoid-induced myopathy is well-established and involves multiple mechanisms including accelerated protein catabolism, impaired protein synthesis, mitochondrial dysfunction, and muscle fiber atrophy, particularly of type II fast-twitch fibers [6,7,12]. While these mechanisms have been extensively studied in overt Cushing’s syndrome and in patients receiving exogenous glucocorticoid therapy, their clinical relevance in MACS has been less clear. Our findings provide objective evidence that even mild, autonomous cortisol excess has measurable negative effects on skeletal muscle function, and importantly, that surgical correction can reverse these effects.

The observed 1.2 kg improvement in grip strength in the surgical group, though modest in absolute terms, represents a clinically meaningful change. Studies have established that grip strength changes of 5–6 kg are associated with decreased disability and mortality risk in older adults [13,14]. Furthermore, the 5% improvement we observed aligns with minimally important differences reported for grip strength in various populations [15]. The fact that conservatively managed patients experienced declining grip strength over 6 months further emphasizes the progressive nature of MACS-related muscle impairment and the potential benefit of surgical intervention.

Clinical Significance and Minimal Clinically Important Difference (MCID):

While the observed grip strength improvement of approximately +1.2 kg (+4.7%) is statistically significant within the surgical group, consideration of the minimal clinically important difference (MCID) is warranted. Published MCID estimates for grip strength in adult populations range from 5.0 to 6.5 kg, suggesting that our observed improvement falls below these established thresholds for individual-level clinical significance. However, several factors warrant reconsideration: (1) the MCID concept typically applies to symptomatic patients seeking treatment; (2) our cohort exhibits relatively preserved baseline grip strength, limiting the potential for large absolute improvements; and (3) the minimal detectable change (MDC), which represents measurement error and true variability in the test itself, is substantially smaller than the MCID (estimated MDC ≈ 0.8 kg), and our observed change exceeds this threshold. From a health trajectory perspective, stabilization and improvement of grip strength versus progressive decline may carry clinical importance for long-term disability risk and functional independence.

Limitations of Distal Muscle Strength Assessment: Glucocorticoid-induced myopathy classically presents with selective atrophy of Type II (fast-twitch) skeletal muscle fibers, affecting proximal muscles (quadriceps, gluteus maximus, hip flexors) preferentially over distal muscles. Our study focused on hand grip strength, a distal measure, which may not fully capture cortisol-related myopathy. Proximal muscle weakness (difficulty rising from a chair, climbing stairs, weakness with hip and knee extension) may be more specific for steroid myopathy and could represent a more sensitive outcome. Future studies should incorporate validated proximal muscle assessments such as the Chair Stand Test (repeated chair stand time), stair climbing time, or lower extremity dynamometry to comprehensively evaluate the full spectrum of glucocorticoid effects on skeletal muscle. The inclusion of such proximal measures alongside grip strength would strengthen evidence regarding the muscle phenotype of MACS and its response to surgical intervention.

Our results complement and extend existing data on post-adrenalectomy outcomes in MACS. Previous studies have demonstrated improvements in cardiometabolic parameters, bone mineral density, and quality of life following surgery [16,17]. However, systematic assessment of muscle strength using validated instruments has been largely absent from the literature. Grip strength measurement offers several advantages as a functional outcome: it is quick, requires minimal equipment, has excellent reliability, and correlates with overall muscle strength and functional capacity [8,9]. In clinical practice, documenting objective muscle weakness before surgery may help identify patients most likely to experience functional benefit from adrenalectomy.

Postoperative Glucocorticoid Management and Muscle Recovery: An important consideration in interpreting the post-adrenalectomy muscle strength improvements is the management of glucocorticoid replacement. Five patients in our surgical cohort required prolonged glucocorticoid support (maximum 45 days) to manage secondary adrenal insufficiency and withdrawal syndrome symptoms. Glucocorticoid withdrawal syndrome—characterized by fatigue, myalgias, and arthralgias—can transiently impair muscle function and physical performance. The timing of hormone replacement and the individual trajectory of HPA axis recovery varied among patients. Despite this heterogeneity in perioperative glucocorticoid management, the surgical group demonstrated consistent improvements in grip strength, suggesting that cessation of autonomous cortisol excess yields functional benefit that outweighs the temporary negative effects of glucocorticoid withdrawal. This observation emphasizes the importance of close perioperative monitoring and individualized glucocorticoid tapering to minimize withdrawal symptoms while supporting HPA axis recovery.

The lack of significant within-group improvement in gait speed in our surgical cohort (−0.03 ± 0.06 s, *p* = 0.180), despite significant improvements in grip strength, warrants discussion. Gait is a complex motor task involving multiple muscle groups (proximal leg muscles, core stabilizers), balance, proprioception, coordination, and neurological integration; it is thus less specific to hand grip than dynamometry alone. Several explanations may account for the gait findings: (1) 6 months may be insufficient time to observe meaningful changes in this higher-order functional measure; (2) the degree of cortisol excess in MACS may not be severe enough to cause substantial gait impairment; and (3) age-related decline and musculoskeletal comorbidities may mask potential improvements. Notably, the conservative group exhibited a statistically significant worsening of gait speed (+0.04 ± 0.06 s, *p* = 0.002), suggesting progressive functional deterioration over time.

From a clinical practice perspective, our findings suggest that objective muscle strength assessment should be incorporated into the evaluation of MACS patients being considered for surgery. Current guidelines from the European Society of Endocrinology and AACE/AAE recommend individualized decision-making for MACS management, considering factors such as comorbidities, age, tumor characteristics, and patient preferences [1,5]. Our data support adding documented muscle weakness measured objectively with dynamometry to the list of factors that may favor surgical intervention, especially in younger, symptomatic patients with long life expectancy.

The practical implications are straightforward: hand dynamometers are portable, affordable (typically $50–200 USD), require minimal training to use, and provide reliable measurements in under 2 min. For clinicians managing MACS patients, baseline grip strength assessment can help objectively document functional impairment and track post-operative recovery. Patients presenting with grip strength substantially below age- and sex-matched norms may represent a subgroup particularly likely to benefit functionally from adrenalectomy.

Baseline age differences warrant mention as a potential limitation. The surgery group was numerically younger than the conservative group, and age is a key determinant of muscle mass and strength trajectories. In a modest-sized retrospective cohort, such baseline heterogeneity may contribute to observed differences in functional change over time independent of cortisol normalization.

### 4.1. Study Limitations

Design and Randomization:

First, this was a non-randomized comparative cohort study with group assignment based on clinical indication and patient preference rather than randomization. Unmeasured confounders may have influenced outcomes. Accordingly, our findings should be interpreted as associative rather than causal.

Sample Size and Statistical Power:

Second, the sample size was modest (n = 40: n = 15 surgery, n = 25 conservative). This limits precision for smaller effect sizes and precludes extensive subgroup analyses by age, BMI, or comorbidities.

Follow-up Duration:

Third, our follow-up period of 6 months may be insufficient to capture the full extent of muscle strength recovery or to assess durability of functional improvements. Extended follow-up studies are warranted.

Confounding Variables Not Controlled:

Fourth, we did not prospectively measure or control for several variables that could influence muscle strength independently of cortisol levels, including physical activity, nutritional status, vitamin D supplementation, rehabilitation participation, neuropathy severity, joint disease burden, and inflammatory markers.

Assessment of Distal vs. Proximal Muscle Strength:

Fifth, our assessment focused on distal muscle strength (hand grip). We did not assess proximal muscle function via Chair Stand Test, stair climbing time, or lower extremity dynamometry, which may underestimate glucocorticoid-related myopathy.

Gait Speed Measurement:

Sixth, gait performance was assessed using a 1-m walk test rather than more standardized measures (4-m walk test [4MWT] or 6-min walk test [6MWT]). The 1-m distance is brief, which increases relative measurement error and may not fully capture sustained walking ability.

Postoperative Glucocorticoid Management Details:

Seventh, while we documented whether glucocorticoid replacement was provided until HPA axis recovery, we did not systematically quantify detailed tapering schedules or withdrawal symptom severity for individual patients, and these factors may transiently affect functional outcomes.

Clinical Significance and MCID:

Eighth, while the observed grip strength improvement exceeds the minimal detectable change (MDC ≈ 0.8 kg), it remains below some published minimal clinically important difference (MCID) thresholds (5.0–6.5 kg), highlighting the need for larger studies with responder analyses.

Sex and Demographics:

Finally, the study population was predominantly female (85%), limiting generalizability to male patients.

Third, our follow-up period of 6 months may be insufficient to capture the full extent of muscle strength recovery or to assess long-term outcomes. Glucocorticoid-induced myopathy may take 6–12 months or longer to fully reverse after the underlying cause is corrected [18]. Extended follow-up studies are warranted to determine whether muscle strength continues to improve beyond 6 months and whether early gains are sustained long-term.

Fourth, we did not control for potential confounders such as physical activity levels, nutritional status, or use of physical therapy, which could influence muscle strength independently of cortisol levels. Future studies incorporating structured exercise interventions or rehabilitation protocols could help delineate the relative contributions of cortisol normalization versus physical training to muscle recovery.

Finally, our study population was predominantly female (85%), reflecting the typical demographics of adrenal incidentaloma cohorts, but limiting generalizability to male patients. Sex-specific analyses were not feasible given the small number of male participants.

### 4.2. Clinical Implications and Future Directions

Despite these limitations, our study has important clinical implications. Hand grip dynamometry ought to be regarded as a significant enhancement to the pre-operative assessment of MACS patients. Clinicians should consider documenting baseline grip strength, particularly in patients reporting muscle weakness or fatigue, as this may help stratify patients for surgical benefit and provide objective outcome measures for post-operative follow-up.

Future research should focus on several key areas: (1) randomized controlled trials comparing surgery versus observation, though acknowledging the practical and ethical challenges; (2) longer-term follow-up studies (≥12–24 months) to assess sustained functional benefits; (3) identification of baseline predictors of muscle strength improvement post-adrenalectomy; (4) assessment of whether adjunctive interventions (e.g., resistance exercise, protein supplementation) enhance muscle recovery; and (5) cost-effectiveness analyses incorporating functional outcomes alongside traditional metabolic endpoints.

## 5. Conclusions

This retrospective comparative cohort study suggests that adrenalectomy may be associated with improvement in objective muscle strength in patients with MACS, alongside biochemical resolution of cortisol excess. In contrast, conservative management was associated with a decline in muscle strength over 6 months. Hand grip dynamometry is a simple, reliable, and inexpensive office-based tool that may help document functional impairment and monitor change over time in MACS. Given the retrospective non-randomized design and modest sample size, these findings should be interpreted cautiously, and prospective studies are needed to confirm causality and to define clinically meaningful responder thresholds.

## Figures and Tables

**Table 1:** Baseline Characteristics of Study Participants.

Characteristic	Surgery Group (*n* = 15)	Conservative Group (*n* = 25)
Demographics		
Age (years)	53.6 ± 9.4	57.8 ± 9.1
Female, n (%)	13 (87%)	21 (84%)
BMI (kg/m^2^)	34.7 ± 10.1	33.2 ± 6.5
Comorbidities, n (%)		
Diabetes mellitus	6 (40%)	10 (40%)
Hypertension	9 (60%)	16 (64%)
Cardiovascular disease	8 (53%)	9 (36%)
Hyperlipidemia	5 (33%)	9 (36%)
Osteoporosis	5 (33%)	5 (20%)
Hormonal Parameters		
Morning cortisol (µg/dL)	14.03 ± 4.83	14.80 ± 5.01
ACTH (pg/mL)	3.95 ± 1.47	8.25 ± 8.56
1-mg DST (µg/dL)	4.05 ± 1.44	3.88 ± 3.20
Muscle Strength & Function		
Right hand grip (kg)	25.49 ± 6.31	23.09 ± 8.14
Left hand grip (kg)	24.83 ± 6.07	22.16 ± 7.10
Gait speed (seconds)	1.17 ± 0.20	1.28 ± 0.26
DHEA-S (ng/mL)	59.58 ± 50.16	68.38 ± 49.98

Data are presented as mean ± SD or n (%). BMI, body mass index; ACTH, adrenocorticotropic hormone; DST, dexamethasone suppression test.

**Table 2:** Changes in Muscle Strength and Gait Speed from Baseline to 6 Months.

Parameter	Surgery Group (n = 15)	*p*-Value (Within Group)	Conservative Group (n = 25)
Right Hand Grip Strength (kg)			
Baseline	25.49 ± 6.31		23.09 ± 8.14
6-month	26.68 ± 6.53		22.69 ± 8.07
Change	+1.19 ± 0.64	<0.001	−0.40 ± 0.25
% Change	+4.7%		−1.7%
Left Hand Grip Strength (kg)			
Baseline	24.83 ± 6.07		22.16 ± 7.10
6-month	25.98 ± 6.13		21.88 ± 6.99
Change	+1.15 ± 0.49	<0.001	−0.28 ± 0.28
% Change	+4.6%		−1.3%
Gait Speed (seconds for 1 m)			
Baseline	1.17 ± 0.20		1.28 ± 0.26
6-month	1.14 ± 0.19		1.32 ± 0.26
Change	−0.03 ± 0.07	0.180	+0.04 ± 0.06
1-mg DST (µg/dL)			
Baseline	4.05 ± 1.44		3.88 ± 3.20
6-month	1.01 ± 0.34		Not assessed
Change	−3.04 ± 1.57	<0.001	

Data are presented as mean ± SD. Within-group *p*-values from paired *t*-test. DST, dexamethasone suppression test. Negative change in gait speed indicates improvement (faster walking).

**Table 3:** Baseline Comparison Between Surgical and Conservative Groups.

Variable	Surgical (n = 21)	Conservative (n = 19)	*t*	*p*-Value
Age (years)	54.6 ± 8.9	58.1 ± 9.7	−1.167	0.251
Grip Strength, baseline (kg)	26.50 ± 6.71	21.21 ± 7.55	2.348	0.024 *
1-Meter Walk Speed, baseline (s)	1.146 ± 0.183	1.327 ± 0.269	−2.427	0.020 *

Values are mean ± SD. Independent samples *t*-test. * *p* < 0.05.

**Table 4:** Within-Group Pre- and Post-Treatment Comparison (Paired *t*-test).

Outcome	Group	Pre-Treatment Mean ± SD	Post-Treatment Mean ± SD	Change (Post − Pre)	*t*	*p*-Value
Grip Strength (kg)	Surgical	26.50 ± 6.71	27.22 ± 6.37	+0.719 ± 0.941	−3.501	0.002 *
	Conservative	21.21 ± 7.55	20.83 ± 7.42	−0.382 ± 0.267	6.242	<0.001 *
1-Meter Walk (s)	Surgical	1.146 ± 0.183	1.143 ± 0.150	−0.004 ± 0.070	0.231	0.820
	Conservative	1.327 ± 0.269	1.365 ± 0.238	+0.038 ± 0.064	−2.595	0.018 *

Values are mean ± SD. * *p* < 0.05 (paired t-test). Change = Post − Pre. Positive change indicates grip strength improvement; negative walk time indicates faster (improved) gait.

**Table 5:** Between-Group Comparison of Change Scores (Surgical vs. Conservative).

Outcome	Surgical Δ Mean ± SD	Conservative Δ Mean ± SD	*t*	*p*-Value	Cohen’s d
Grip Strength (kg)	+0.719 ± 0.941	−0.382 ± 0.267	4.919	<0.001 *	1.56 (large)
1-Meter Walk (s)	−0.004 ± 0.070	+0.038 ± 0.064	−1.921	0.063	−0.62 (medium)

Δ = Post-treatment − Pre-treatment. * *p* < 0.05. Independent samples *t*-test following normality confirmation (Shapiro-Wilk: Grip Surgical *p* = 0.360, Conservative *p* = 0.899; Walk Surgical *p* = 0.110, Conservative *p* = 0.056). Mann-Whitney U: Grip U = 335, *p* < 0.001; Walk U = 116.5, *p* = 0.063. Cohen’s d: small = 0.2, medium = 0.5, large ≥ 0.8.

**Table 6:** ANCOVA: Post-Treatment Outcomes Adjusted for Baseline Value and Age.

Outcome	Predictor	β	SE	*t*	*p*-Value	Model R^2^
Grip Strength Post (kg)	Intercept	23.506	0.162	145.38	<0.001	0.993 (n = 40)
	Group (Surgical vs. Conservative)	1.299	0.231	5.618	<0.001 *	
	Baseline Grip Strength	0.964	0.015	62.34	<0.001	
	Age (years)	0.002	0.012	0.148	0.883	
1-Meter Walk Post (s)	Intercept	1.291	0.013	96.33	<0.001	0.944 (n = 38)
	Group (Surgical vs. Conservative)	−0.073	0.020	−3.703	<0.001 *	
	Baseline Walk Speed	0.836	0.042	20.11	<0.001	
	Age (years)	−0.0004	0.001	−0.397	0.694	

β = unstandardized regression coefficient. SE = standard error. * *p* < 0.05. Model: Post-treatment outcome ~ Group + Baseline Value + Age (covariates centered). Negative β for Group in walk model indicates lower (faster) post-treatment walk time in the surgical cohort after adjustment.

## Data Availability

The data presented in this study are available on request from the corresponding author due to privacy and ethical restrictions.
